# Intraocular Plasmablastic Lymphoma in a HIV Patient

**DOI:** 10.1155/2017/7693149

**Published:** 2017-08-08

**Authors:** Carolline Fontes Alves Mariano, Glauce Lunardelli Trevisan, Antonio Augusto Velasco e Cruz, Fernando Chahud

**Affiliations:** ^1^Department of Pathology and Forensic Medicine, School of Medicine of Ribeirão Preto, University of São Paulo, Ribeirão Preto, SP, Brazil; ^2^Department of Ophthalmology, Otorhinolaryngology and Head and Neck Surgery, School of Medicine of Ribeirão Preto, University of São Paulo, Ribeirão Preto, SP, Brazil

## Abstract

Plasmablastic lymphoma (PBL) is a rare B-cell lymphoma occurring mainly in HIV patients. The tumor frequently involves extranodal sites such as the oral cavity, nasal cavity, gastrointestinal tract, skin, and lungs. The neoplastic cells are characterized by a plasmablastic appearance and typical immunophenotype that indicates plasma cell differentiation. Herein, we report a case of intraocular involvement by plasmablastic lymphoma in a HIV patient with a long history of cytomegalovirus retinitis and loss of vision. After an evisceration performed to control the blind, painful eye a diagnosis of PBL was made by examining the eye contents. Two months later, a biopsy of cervical lymph node revealed nodal lymphoma of the same type. To the best of our knowledge, this is the second case of intraocular PBL reported in English literature.

## 1. Introduction

PBL was first described by Delecluse et al. [1] in 1997, occurring preferentially in the oral cavity of HIV-infected patients. It is a specific type of diffuse large B-cell lymphoma (DLBL) with plasmablastic morphology, high proliferative index, and plasmacytoid immunophenotype (positive for CD138 and MUM-1 and negative for CD20). Most PBL are extramedullary, aggressive tumors associated with HIV infection [2] with a median age of presentation of 38 years and a male predominance of 7 : 1 [3]. The tumor has also been reported in immunocompetent and posttransplant patients [4]. The pathogenesis of PBL HIV-associated is poorly understood. However, it is suggested that Epstein-Barr virus infection and* MYC* dysregulation mediated by translocation or amplification [5] could allow plasmablasts to escape apoptosis.

Primary intraocular lymphoma (PIOL) is a variant of primary central nervous system lymphoma in which lymphoma cells are initially present only in the eyes without evidence of disease in the brain or cerebrospinal fluid [6]. Although rare, the majority of PIOL are DLBL.

Secondary intraocular lymphoma arises outside the central nervous system and metastasizes to the eye. Most of them involve the uveal tract and DLBL is also the most frequent histologic subtype [7].

The aim of this study is to report a case of secondary intraocular PBL in a HIV patient.

## 2. Case Report

A 38-year-old patient was admitted at the oculoplastic service complaining of a blind, painful eye. On physical examination, there was no light perception in the right eye (OD) and normal vision acuity (20/20) in the left eye (OS). Biomicroscopy disclosed a scleral perforation in OD with loss of uveal tissue. The patient was a known case of HIV infection who was undergoing highly active antiretroviral therapy (HAART). One year and a half before consultation he complained of loss of vision in OD. At that time ocular fundus examination showed diffuse retinitis diagnosed as cytomegalovirus infection and treated with ganciclovir.

Due to the painful blind eye the patient underwent evisceration of the affected eye.

## 3. Material and Methods

**Figure 1:**
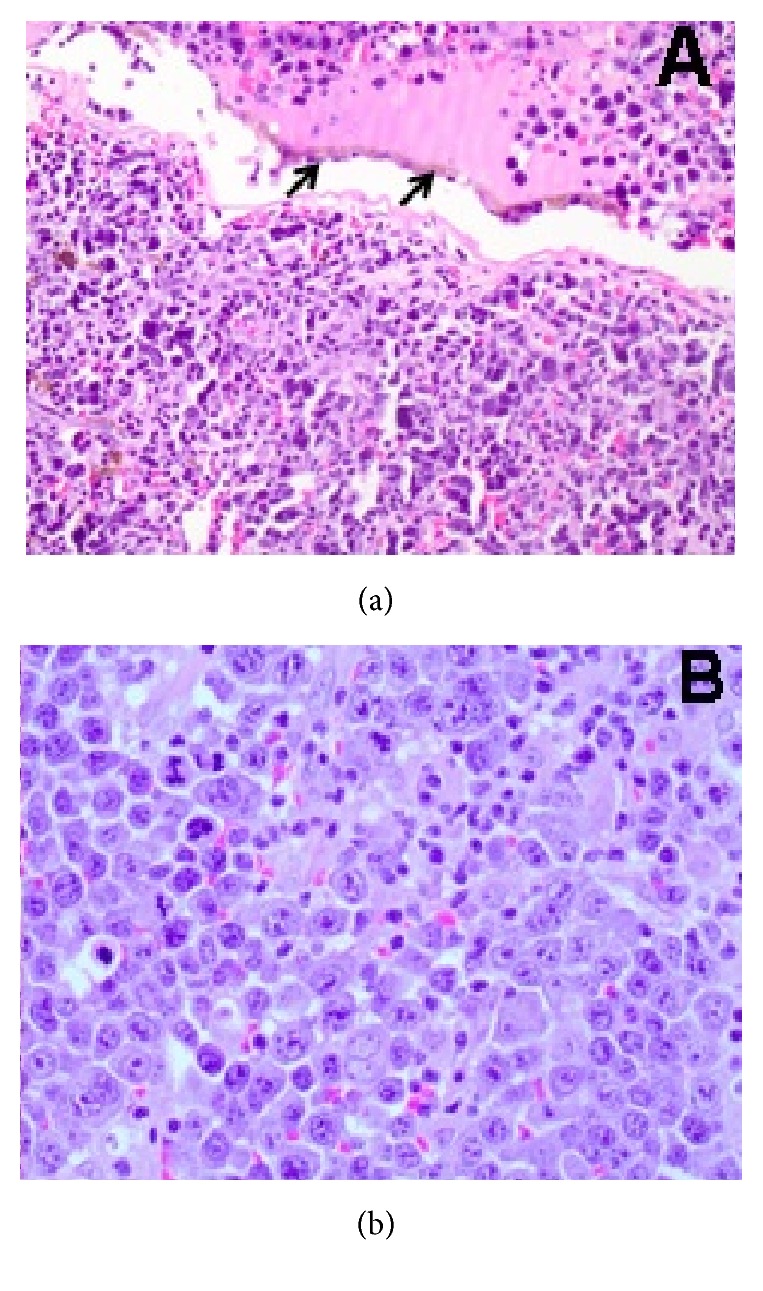
(a) Diffuse infiltration of the choroid and replacement of the retina by tumor cells (H&E 200x; arrows: Retinal Pigment Epithelium) with a plasmablastic appearance ((b) H&E, 400x).

The microscopic examination of the eye contents revealed a diffuse lymphoid tumor infiltrating the choroid and totally replacing the retina (Figure 1(a)) with plasmablastic morphology composed of large cells with abundant basophilic cytoplasm, oval eccentric nuclei, and evident nucleoli (Figure 1(b)). There were also frequent necrotic foci.

**Figure 2:**
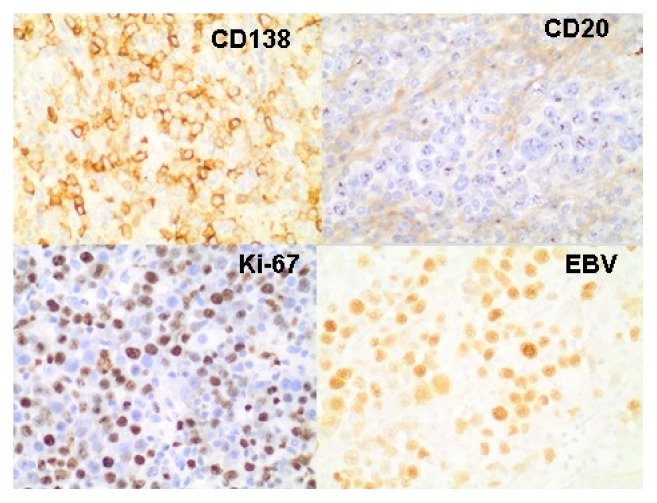
The immunohistochemistry and CISH studies revealed tumors cells positive for CD138 and EBV and negative for CD20. The proliferation index (Ki-67) was around 80%.

The immunohistochemistry study showed positivity of the neoplastic cells for CD38, CD138 (Figure 2), BOB-1, Oct-2, and MUM-1 and negativity for CD45, CD20, CD79a, Pax-5, and CD56. The proliferative index (Ki-67) was around 80%. The chromogenic in situ hybridization (CISH) for Epstein-Barr virus (EBV) was positive (Figure 2). Based on these findings a diagnosis of plasmablastic lymphoma was made.

By the time of the evisceration, blood sample examination showed a CD4 T-cell count of 325 cells/mm^3^ and 82 HIV copies/ml (log 1,914).

Shortly after the diagnosis the patient complained of cervical nodule and a biopsy revealed PBL in lymph node.

The patient was treated with chemotherapy but the lymphoma relapsed 2 years later and the patient died of bacterial pneumonia.

## 4. Discussion

Intraocular lymphomas can occur as primary or secondary lesions. The secondary lesions usually involve the uveal tract, mainly the choroid. A recent study of a large series of choroidal lymphomas showed that 31% of the patients with choroidal lymphoid lesions had known systemic lymphoma at initial presentation or were diagnosed with systemic lymphoma shortly after the initial presentation [7]. This study has also shown that among the cases with secondary intraocular involvement only one patient had the diagnosis of PL. Our patient was also diagnosed with systemic lymphoma shortly after the initial diagnosis of intraocular lymphoma. Interestingly, at that time, there was no hypothesis of eye tumor since the blind, painful eye was attributed to CMV retinitis related complications.

Involvement of ocular adnexa by PBL, although rare, has been more frequently reported when compared to intraocular lesions. A few cases of PBL have been reported involving the orbit [8, 9]. Most of the patients had also systemic involvement at the time of the orbital findings.

At the time of diagnosis our patient was 38 years old. This is in accordance with the study published by Castillo et al. [3] in which the mean age at presentation of PBL in HIV-positive patients was 39 years. The morphological findings and the immunophenotype were both typical of PL. The tumor was composed of large cells with plasmablastic appearance and showed positivity for CD38, CD138, BOB-1, Oct-2, and MUM-1 and negativity for CD45, CD20, CD79a, and PAX-5. In addition, the proliferation index was very high (80%) and Epstein-Barr virus (EBV) was detected by CISH.

The pathogenesis of PBL is poorly understood and complex and many factors such as HIV-related immunodeficiency, genetic cellular abnormalities, coinfecting oncogenic viruses, and chronic immune activation, including CMV infection, play significant roles [5]. In a study with fifty HIV patients who developed tumors with plasmablastic lymphomas, the median CD4-positive count was 206 cells/mm^3^ (range, 5–683 cells/mm^3^) and the median viral load at presentation was 261,560 copies/mL (range, from undetectable to 4.7 million copies/mL) [10]. Both the CD4-positive cell count and the viral load of our patient were within the limits found by the authors of this study. Despite treatment, the patient had a poor survival time (2 years), indicating the aggressive course of the disease as reported in the literature [10, 11].

In our case, even though the initial diagnosis was made by examining the eye tissues, it represents a secondary involvement of the eye since systemic lymphoma was diagnosed shortly after. As far as we know, this is the second case of intraocular involvement by PBL reported in English literature.
